# Artificial Intelligence in Tetralogy of Fallot: From Prenatal Diagnosis to Lifelong Management: A Narrative Review

**DOI:** 10.3390/bioengineering12121349

**Published:** 2025-12-10

**Authors:** Tiziana Fragasso, Davide Passaro, Alessandra Toscano, Antonio Amodeo, Alberto Eugenio Tozzi, Giorgia Grutter

**Affiliations:** 1 Cardiac Intensive Care Unit, Bambino Gesù Children’s Hospital, IRCCS, 00165 Rome, Italy; 2 Department of Statistical Sciences, Sapienza University of Rome, 00185 Rome, Italy; 3 Perinatal Cardiology Unit, Bambino Gesù Children’s Hospital, IRCCS, 00165 Rome, Italy; 4 Heart Failure, Transplantation, Cardiorespiratory Mechanical Assistance Unit, Bambino Gesù Children’s Hospital, IRCCS, 00165 Rome, Italygiorgia.grutter@opbg.net (G.G.); 5 Preventive and Predictive Medicine Unit, Bambino Gesù Children’s Hospital, IRCCS, 00165 Rome, Italy

**Keywords:** artificial intelligence, congenital heart disease, Tetralogy of Fallot, predictive medicine, long-term follow-up, digital twins, machine learning

## Abstract

Artificial intelligence (AI) is rapidly transforming cardiovascular medicine, with profound implications for congenital heart disease (CHD). Tetralogy of Fallot (ToF), the most common cyanotic disease, requires lifelong surveillance and complex management because of late complications such as pulmonary regurgitation, arrhythmias, and right ventricular dysfunction. This review synthesizes current evidence on AI applications across the continuum of ToF care—from prenatal diagnosis to adulthood follow-up. We examine advances in imaging, perioperative planning, intraoperative monitoring, intensive care, and long-term surveillance, including wearable and implantable technologies. Machine learning (ML), deep learning (DL), and natural language processing (NLP) are revolutionizing diagnostic accuracy, risk stratification, surgical decision-making, and personalized long-term care. The future lies in the integration of multimodal data, including imaging, electronic health records (EHRs), genomic information, and continuous monitoring, to support precision medicine. Challenges remain regarding dataset limitations, interpretability, regulatory standards, and ethical concerns. Nevertheless, ongoing innovation and collaboration between clinicians, engineers, and regulators promise a new era in congenital cardiology. By embedding AI throughout the patient journey, healthcare systems may improve outcomes and quality of life for individuals with ToF.

## 1. Introduction

Tetralogy of Fallot (ToF) accounts for 5–7% of all congenital heart defects and is the most common cyanotic lesion beyond infancy [1]. Advances in surgical techniques and perioperative care have dramatically improved survival, with more than 90% of patients now reaching adulthood [2]. Nonetheless, ToF remains a lifelong clinical challenge, as patients continue to be at risk for both early and late complications, including pulmonary regurgitation, progressive right ventricular (RV) dilatation, arrhythmias, sudden cardiac death, and the need for reinterventions [2].

The annual financial burden of complex congenital heart disease (CHD) is strikingly high, with per-patient costs averaging nearly $50,000, almost half of which is borne directly by families [3]. Importantly, 31% of lifetime costs are sustained within the first five years of life, amounting to an average of $650,000 per child, including about $190,000 in out-of-pocket expenses for families. This early concentration of costs places extraordinary strain on caregivers, who often reduce working hours, with an estimated cumulative productivity loss for parents and patients of roughly one month per year. Compared with more common cardiac conditions, the disproportionate and lifelong nature of these costs underscores the urgent need for greater investment in research, innovation, and institutional support programs to alleviate the economic and human toll of complex CHDs.

In parallel, artificial intelligence (AI) is rapidly emerging as a transformative tool in medicine. As of June 2025, the U.S. Food and Drug Administration had cleared 1016 AI-enabled medical algorithms, of which 104 were in cardiology and 777 in radiology—nearly doubling within just two years [4,5]. Machine learning (ML) can identify hidden patterns across multimodal datasets by integrating imaging, hemodynamic, genomic, and wearable-derived data, thereby uncovering clinically relevant associations, predicting patient outcomes, and supporting personalized decision-making that would be difficult to achieve with traditional analytic approaches. Beyond its clinical applications, AI also exerts a significant impact on healthcare economics by optimizing resource allocation, reducing diagnostic and therapeutic inefficiencies, and enabling the earlier detection of complications—factors that could ultimately lower hospitalization rates and long-term costs for public health systems.

This review provides a comprehensive synthesis of how AI can support patient care across all stages of ToF management, from the prenatal period to adulthood, supporting diagnosis, treatment, and individualized follow-up. Specifically, we examine:

- the role of AI in prenatal and perinatal care;
- applications in perioperative management and early postoperative care;
- the contribution of AI to long-term follow-up;
- opportunities arising from data integration and predictive modeling;
- challenges, limitations, and future perspectives for implementing AI in congenital cardiology.

The goal is to provide clinicians and researchers with a practical framework to understand the current state-of-the-art, the potential benefits, and the limitations of AI in ToF, thereby fostering the development of innovative strategies that can be effectively integrated into clinical practice. This review integrates current knowledge from fetal to adult care, offering a roadmap for the role of AI in transforming ToF management. Most of the relevant ToF-related AI studies described in this review are summarized in Table 1, highlighting for each model the input modality, training and validation datasets, clinical outcome, performance metrics, and the presence of an external validation cohort or clinical benchmark.

**Figure 1:**
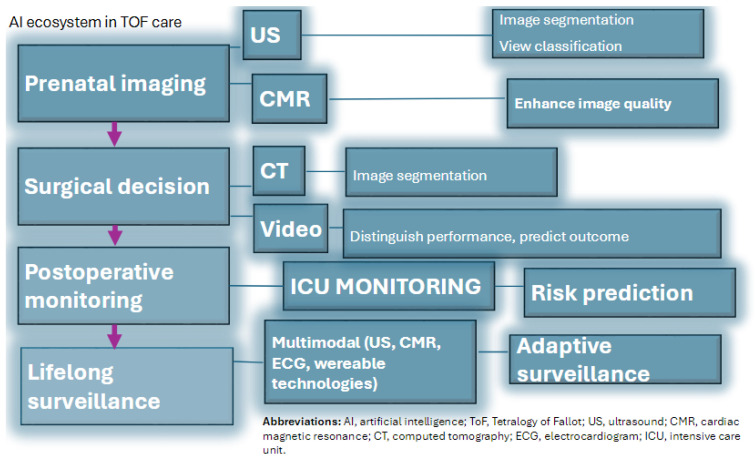
Schematic overview of the TOF AI ecosystem.

Figure 1 provides a schematic overview of the ToF AI ecosystem, illustrating the data flow from prenatal diagnosis through surgical management to lifelong follow-up.

### Literature Search Strategy

This narrative review is based primarily on a literature search performed in PubMed/MEDLINE. We searched the database from January 2000 to October 2024 using combinations of the following terms: “Tetralogy of Fallot”, “congenital heart disease”, “artificial intelligence”, “machine learning”, “deep learning”, “neural network”, “radiomics”, “clinical decision support”, and “digital twin”. We included original studies and reviews reporting applications of AI to ToF, or to broader congenital heart disease populations including ToF, across the patient journey (prenatal diagnosis, perioperative management, and long-term follow-up). We restricted the search to human studies published in English. In addition, a complementary non-systematic search using Google Scholar was performed to identify further relevant articles, including by screening the reference lists of key papers.

## 2. Artificial Intelligence in the Prenatal and Perinatal Phase of Tetralogy of Fallot

Early detection of CHD, including ToF, is crucial for optimizing perinatal and postnatal outcomes. Prenatal diagnosis facilitates planned delivery at specialized centers, coordination of neonatal interventions, and early parental counseling. However, fetal cardiac imaging remains challenging because it requires substantial operator expertise and is susceptible to technical limitations [23]. AI can improve the accuracy and consistency of cardiac imaging interpretation, particularly in echocardiography and fetal cardiac magnetic resonance imaging (MRI) [24].

Fetal echocardiography remains the cornerstone of prenatal diagnosis for CHD, including ToF, but it is limited by operator variability and diagnostic ambiguity, especially when distinguishing overlapping anomalies such as ToF and isolated ventricular septal defects (VSDs). Convolutional neural networks (CNNs), a type of deep learning (DL) algorithm specifically designed to process visual data, are increasingly used to enhance diagnostic accuracy by automating image acquisition, chamber segmentation, and disease classification. AI-driven systems trained on large datasets improve inter-operator consistency and diagnostic yield. Arnaout et al. (2021) [6] reported that DL-based models achieved 71% sensitivity and 89% specificity for detecting ToF from prenatal ultrasound, notably improving upon traditional diagnostic rates, with real-world sensitivity for the prenatal detection of ToF reported as low as approximately 50% [25]. Similarly, Yu et al. [7] compared four CNN architectures and found that the weakly supervised data augmentation network (WSDAN) outperformed the others, with an AUC of 0.873 in differentiating ToF from VSD.

Fetal cardiac MRI, although less widely used than echocardiography because of motion artifacts, lack of ECG gating, and complex acquisition protocols, offers superior spatial resolution and tissue characterization, which is particularly valuable in complex CHD. Uus et al. [8,26] developed AI-based frameworks using CNNs and deformable slice-to-volume registration, significantly enhancing image quality and anatomical interpretability in motion-corrupted datasets and allowing for better visualization of cardiac chambers, vessel alignment, and outflow tract morphology.

Among commercially available software solutions, BrightHeart received FDA 510(k) clearance as the first AI software dedicated to prenatal ultrasound of the fetal heart, supporting clinicians in detecting morphological abnormalities suggestive of CHD and reducing inter-observer variability in resource-constrained settings [27]. Other platforms, such as FetalHQ (GE Voluson), also provide rapid AI-driven assessment of fetal heart size, shape, and function in under three minutes; however, variable reproducibility has been observed among operators, influenced by parameter selection, operator experience, and technical factors [28].

## 3. Applications in Perioperative Management and Early Postoperative Care

In this section, we focus on AI applications during the perioperative window, including preoperative planning, intraoperative guidance, and early postoperative/ICU risk prediction. Medium- and long-term follow-up after discharge are discussed separately in Section 4.

### 3.1. Preoperative Risk Assessment and Timing/Type of Repair

AI applications in perioperative and surgical management span from preoperative planning to intraoperative monitoring and anesthetic management. Surgical repair of ToF typically includes closure of the ventricular septal defect (VSD) and relief of right ventricular outflow tract (RVOT) obstruction, yet the perioperative course remains complex because of anatomical heterogeneity, possible coronary anomalies, and the risk of early complications such as arrhythmias, low cardiac output syndrome, and biventricular dysfunction [1].

AI has been applied to cardiac computed tomography (CT) to improve characterization of right ventricular geometry and pulmonary artery anatomy, enabling more precise delineation of the coronary artery (CA) course, which remains a critical factor in surgical planning and decision-making. Barak-Corren et al. (2025) [9] developed a novel image-derived 3D modeling approach to assess CA proximity to the RVOT in patients undergoing transcatheter pulmonary valve replacement (TPVR) with self-expanding valves, revealing that the anatomical relationship between the RVOT and the CAs was dynamic across the cardiac cycle. In this study, CT-derived segmentations of the RVOT and CAs were created using ML, making 3D segmentation more efficient and enabling processing of multiple frames over time in 4D CT angiography, thus providing a dynamic evaluation of the RVOT. Wang et al. (2021) [10] developed the Structurally Optimized Stochastic Pooling Convolutional Neural Network (SOSPCNN), an advanced DL architecture that combines optimized network design with stochastic pooling to improve accuracy and generalization in medical image analysis. They achieved highly accurate CT-based recognition of ToF, outperforming existing state-of-the-art methods. These tools not only improve anatomical comprehension but also reduce variability between operators and institutions.

### 3.2. Intraoperative Decision Support

In the operating theater, two supervised ML models have been developed to support surgeons during pulmonary valve replacement in ToF patients [11]. Using a novel video-based technique called video Kinematic Evaluation (Vi.Ki.E), Lo Muzio et al. recorded and analyzed the motion of the beating right ventricle (RV)—which cannot be properly evaluated by transesophageal echocardiography—during surgery. They trained k-nearest neighbor and support vector machine classifiers on intraoperative videos of the beating RV in 12 ToF patients undergoing pulmonary valve replacement: videos acquired before valve implantation were labelled ‘unhealthy’, and those acquired after valve replacement ‘healthy’. By extracting motion features from 86 high-speed videos and training these supervised models, they achieved very high accuracy (true-positive rates ≥ 95% and AUC ~0.97–0.99) in distinguishing ‘unhealthy’ pre-replacement from ‘healthy’ post-replacement RV function before chest closure. The models also successfully predicted outcomes in additional patients not included in the training set and may help guide real-time surgical decisions. Such systems may be particularly valuable in minimally invasive or robotic-assisted interventions, where direct visualization is limited.

### 3.3. Early Postoperative and ICU Risk Prediction

The early postoperative phase following surgical repair of ToF is a critical period marked by a heightened risk of hemodynamic instability, ventricular dysfunction, arrhythmias, and respiratory complications. Optimal management during this time requires continuous monitoring, rapid interpretation of complex physiological data, and timely therapeutic interventions to identify patients at risk of rapid deterioration.

AI has the potential to support clinicians in risk stratification through predictive modeling. ML algorithms trained on large surgical and imaging datasets can integrate preoperative variables—including laboratory values, echocardiographic data, and clinical scores—to predict adverse outcomes such as arrhythmias, need for prolonged mechanical ventilation, or extracorporeal support. Xi et al. [12] explored risk factors for adverse events and developed ML-based prediction models to forecast the incidence of clinical adverse events after ToF repair. They evaluated five ML algorithms and identified key risk factors such as cardiopulmonary bypass (CPB) time, RVOT pressure gradient, and transannular patch repair. Among all models, logistic regression and Gaussian naïve Bayes performed most consistently, showing good discrimination (i.e., effectively distinguishing patients at higher versus lower risk of postoperative adverse events; see Table 1 for performance metrics), calibration, and clinical utility.

The great innovation behind these approaches is that continuous data streams from multiple bedside sources—electrocardiograms, ventilators, infusion pumps, blood gas analyzers, and multiparameter monitors—are already captured within the ICU/EHR ecosystem and can be analyzed in near real-time. This infrastructure provides a natural substrate for electronic clinical decision support systems (CDSSs) and early warning tools that use routinely collected signals to detect subtle signs of impending deterioration, such as changes in cardiac output, rising lactate levels, or evolving arrhythmias, and to trigger timely alerts for the clinical team.

In this context, García-Canadilla et al. [29] described CORTEX, a rule-based (not ML-driven) early warning system that uses six age- and physiology-adjusted vital signs (heart rate, respiratory rate, SpO_2_, systolic and diastolic blood pressure, and temperature) to stratify the risk of clinical deterioration in children after cardiac surgery. In their 1-year pilot study, CORTEX scores increased significantly up to four hours before adverse events, enabling earlier intervention. Its implementation was associated with a 7% reduction in ICU length of stay and an 8% reduction in overall hospital stay, corresponding to approximately €138,000 in total annual hospital savings (not per patient). Importantly, the authors outline the development of an advanced, machine-learning–based upgrade that will integrate more than 50 clinical variables from continuous monitoring, laboratory data, medications, and imaging to enhance predictive performance.

## 4. The Contribution of Artificial Intelligence to Long-Term Follow-Up of Tetralogy of Fallot

This section addresses the medium- and long-term management of repaired TOF, beyond the early postoperative period, including pulmonary regurgitation, RV dilation and dysfunction, arrhythmias, and sudden cardiac death.

### 4.1. Medium-Term Outcomes After Initial Repair

Traditional surveillance protocols—based on rigid, guideline-defined intervals—often fail to reflect interindividual differences in disease progression. AI offers transformative potential to overcome these limitations through dynamic, data-driven monitoring and predictive modeling.

One of the most promising frontiers is the shift from fixed follow-up intervals to AI-driven adaptive surveillance. ML models trained on multimodal longitudinal datasets—integrating imaging, electrocardiography, Holter data, biomarkers, and electronic health records (EHRs)—can predict when each patient is at greatest risk for complications. Such models allow clinicians to anticipate adverse events rather than respond reactively. In adults with repaired ToF, Ishikita et al. [13] developed ML models combining clinical and MRI-derived parameters to predict major adverse cardiovascular events (MACE), thereby informing surgical timing and long-term surveillance strategies. In a more recent study, Ishikita et al. [14] compared 5-year MACE prediction in repaired ToF using either a validated ML model or a multidisciplinary team of adult congenital heart disease experts. Model performance was comparable to that of highly experienced physicians (>20 years), and the incorporation of ML improved risk prediction among less experienced clinicians. Importantly, such adaptive surveillance frameworks should be understood as decision support tools rather than autonomous systems. The model’s role is to highlight periods of increased predicted risk, prompting clinicians to consider additional echocardiography, CMR, Holter monitoring, or biomarker testing within predefined protocols. All diagnostic procedures remain under physician prescription, and no validated branching decision tree for adaptive follow-up exists in repaired TOF.

Earlier, Samad et al. [15] showed that baseline cardiac MRI features could predict subsequent RV functional deterioration, highlighting the potential for trajectory-based scheduling. More recently, Mayourian et al. [16] applied DL to electrocardiography (ECG) signals, outperforming traditional QRS-duration metrics in predicting mortality.

Collectively, these findings emphasize that AI-based dynamic follow-up could replace static, one-size-fits-all models with personalized risk trajectories.

Cardiac MRI has so far been the most extensively explored imaging modality in AI research and remains the cornerstone of long-term follow-up in ToF. From acquisition to reconstruction and post-processing, DL algorithms have been shown to improve speed, accuracy, and reproducibility. Most models have been trained and validated using cardiac MRI datasets of structurally normal hearts or cases with acquired cardiac disease and are therefore not well-suited to handle cases with congenital cardiac disease such as ToF.

Tilborghs et al. [17] developed and validated a fully automatic segmentation and quantification approach for LV and RV, including RV mass, in patients with repaired TOF with a superior performance in RV quantification compared to commercial software.

Govil et al. [18] demonstrated a DL pipeline for automated view classification, slice and phase selection, landmark localization, and myocardial segmentation, enabling end-to-end cardiac shape modeling in repaired ToF. This reduced the time needed to obtain a single cardiac shape model to 5.1 ± 2.8 min, compared with 60–90 min per manual model by an expert analyst. Leiner et al. [24] outlined how ML impacts the full continuum of cardiac MRI—from patient scheduling and image acquisition to diagnostic interpretation and prognostic modeling. Integration of ML within cardiac MRI workflows enables accelerated image reconstruction, artifact correction, and prognostic insight by linking imaging features with clinical and genetic data. Nevertheless, challenges persist, including limited data generalizability across scanners and institutions, data scarcity in rare conditions such as CHD, and the “black-box” nature of DL algorithms [19]. Ongoing work in explainable AI (XAI) seeks to increase transparency and clinician trust, promoting responsible implementation in congenital cardiology.

### 4.2. Long-Term Surveillance and Adult Congenital Outcomes

AI also extends ToF care beyond the hospital setting through continuous physiological surveillance. Smartwatches, patches, and implantable devices generate continuous streams of data on heart rate, rhythm, oxygen saturation, and activity. ML algorithms can analyze these data in real-time to detect subclinical arrhythmias or early hemodynamic compromise, prompting timely telemedicine review and preventing unplanned admissions. Jacquemyn et al. [30] anticipate that decentralized mobile health platforms—integrated with AI-based anomaly detection—will be essential for future ToF management.

Recent work demonstrates the clinical feasibility of these technologies: Umer et al. [31] proposed an IoT-based monitoring framework for heart failure in adults.

The emerging concept of the digital twin represents the ultimate expression of this approach. A digital twin is a dynamic, virtual replica of a patient that integrates clinical, imaging, electrophysiological, and genomic data to simulate disease trajectories and predict responses to interventions [32]. In ToF, patient-specific digital twins could model RV dyssynchrony, guide pulmonary valve replacement timing, and assess the impact of resynchronization therapy [20]. Computational simulations have already shown promise in predicting valve replacement outcomes, quantifying arrhythmia risk, and visualizing long-term remodeling [33].

From an implementation perspective, building a cardiac digital twin is likely to be highly resource-intensive. Outside medicine, Su et al. recently proposed an activity-based costing model for digital twins in manufacturing, with Ref. [34] showing that development costs are driven by data acquisition, IT infrastructure, and specialized personnel, and can range from a few thousand to almost forty thousand pounds even for relatively simple unit-level twins with limited data streams. In pediatrics, Calcaterra et al. [35] described digital-twin systems as projects whose complexity and costs may be comparable to large scientific endeavors such as the Human Genome Project, underscoring that substantial investment will be required before large-scale deployment. Taken together, these data suggest that cardiac and CHD digital twins will demand significant upfront effort and funding, while formal micro-costing and cost-effectiveness analyses specific to congenital heart disease are still lacking and should be a priority for future research. For AI to achieve clinical maturity, integration into the EHR and workflow is crucial. Automated risk dashboards, structured summaries, and clinician alerts could link AI predictions directly with decision support systems. NLP can extract relevant variables from unstructured clinical notes, operative reports, and imaging narratives, thereby reducing manual data entry and making hidden information available to downstream models.

Marelli et al. [21] demonstrated how ML applied to large-scale administrative and clinical datasets can refine the diagnosis of congenital heart disease, highlighting the potential of predictive analytics to uncover patterns that may elude conventional classification. In parallel, Alkan et al. [22] showcased the promise of multi-modal data integration—combining clinical, physiologic, and geometric information—to predict cardiopulmonary exercise outcomes in CHD patients. Together, these studies illustrate how predictive modeling and integrated data streams can not only improve diagnostic accuracy but also anticipate functional trajectories, thereby reinforcing the opportunity for a more proactive and individualized approach to pediatric cardiology care.

## 5. Challenges, Limitations, and Future Perspectives for Implementing AI in Congenital Cardiology

So far, most predictive modeling in congenital cardiology remains at the pre-implementation stage. Despite rapid algorithmic development, only a small fraction of AI tools have reached routine clinical use. Barriers include limited external or prospective validation, poor workflow integration, regulatory friction, unclear return on investment, and slow clinical adoption [36].

Recent systematic reviews in critical care have emphasized that operationalization—the process of moving from proof-of-concept to bedside implementation—requires robust evidence, alignment with clinical workflows, and clear governance structures [36,37]. Expert consensus papers in critical care underscore similar priorities: human-centered design, clinician education, standardized datasets, cross-disciplinary collaboration, and strong institutional governance [38].

Cultural barriers also persist: gaps in clinician AI literacy and, conversely, limited clinical literacy among data scientists; lack of clinician-champions; and workflow disruption that erodes trust. Ethical and legal concerns include bias risks, unclear liability in the clinician–model–vendor triad, uncertain reimbursement, and the need for continuous post-market surveillance.

Beyond technical readiness, ethical challenges remain pivotal. Algorithmic decision-making raises questions of accountability, privacy, and patient autonomy. Grote and Berens [39] argue that ensuring fairness and interpretability is as critical as accuracy when deploying AI in healthcare. Clear frameworks for human oversight and continuous post-market monitoring are essential to maintain trust.

Liability for AI-supported decisions remains one of the most contentious and least resolved issues in healthcare deployment. In most current implementations, AI tools for ToF (e.g., risk scores, image-segmentation pipelines) are classified as decision support systems rather than autonomous decision-makers; accordingly, physicians and institutions still bear the primary responsibility for clinical decisions and for the way AI outputs are used [39,40]. At the same time, manufacturers and software providers may incur product-liability exposure if systems are defective, poorly updated, or marketed without adequate warnings. Recent European initiatives—including the AI Act and proposals for an AI Liability Directive and a revised Product Liability Directive—seek to clarify the obligations of developers, deployers, and clinicians, but a coherent liability framework specific to medical AI is still evolving, and the dedicated AI Liability Directive has recently been withdrawn, leaving a degree of “liability vacuum” to be filled by national tort law and general product-liability rules [41,42,43,44]. Clinicians understandably fear becoming “liability sinks” for opaque algorithms, which in turn dampens adoption. For congenital cardiology and ToF, transparent model documentation, clear role definitions (AI as a second reader or advisory tool), rigorous validation, and institutional policies on documentation of AI use will be essential to balance innovation with legal and ethical accountability.

In the European Union, the Artificial Intelligence Act (Regulation (EU) 2024/1689) [42] introduces a unified, risk-based framework. Most medical imaging and decision support systems will be classified as high-risk, triggering strict requirements for data governance, human oversight, cybersecurity, transparency, and continuous post-market monitoring—aligned with the Medical Device Regulation (MDR) and the In Vitro Diagnostic Regulation (IVDR). These are further reinforced by the NIS2 Directive, the Cyber Resilience Act, the Data Act, and the forthcoming European Health Data Space (EHDS).

In the United States, regulation is more fragmented. The Food and Drug Administration (FDA) oversees Software as a Medical Device (SaMD) pathways, including Predetermined Change Control Plans (PCCPs); the Office of the National Coordinator for Health IT (ONC) enforces interoperability rules; and the National Institute of Standards and Technology (NIST) promotes voluntary risk-management frameworks. A pragmatic takeaway for developers is to treat compliance as a design feature—incorporating documentation, traceability, bias monitoring, human-in-the-loop oversight, and incident reporting from the earliest stages [43].

CHD presents unique challenges for AI deployment. Phenotypes are highly heterogeneous and evolve over decades, while traditional evidence-based medicine struggles with small, underpowered cohorts. As rare conditions, CHD populations are inherently limited in size, but the amount of information that can be extracted from each patient is immense, encompassing multimodal and longitudinal data such as echocardiography, cardiac MRI, catheterization, operative notes, wearables, and genomics—often fragmented across EHRs. Targets such as arrhythmia burden or right-ventricular remodeling are dynamic, complicating labeling and calibration.

This richness of longitudinal data can, however, empower pattern-recognition models. Privacy-preserving federated learning and synthetic datasets may help overcome single-center limitations, especially for ToF surveillance tasks such as pulmonary valve replacement (PVR) timing and arrhythmia prediction. These approaches can transform data scarcity into an opportunity for multimodal learning across institutions.

Cross-cutting barriers are a concern. Data limitations include restricted access to accurately labeled, interoperable datasets; fragmented storage; uncertain ownership; and strict data-protection duties under the European General Data Protection Regulation (GDPR) [40] and the US Health Insurance Portability and Accountability Act (HIPAA) [41].

Beyond the generic issues of data fragmentation and bias, ToF datasets pose specific challenges. First, there is marked class imbalance: even in large institutional archives, the number of ToF patients is small compared with healthy subjects or with more common acquired cardiovascular diseases, which increases the risk of overfitting and unstable performance estimates. Second, “label drift” is intrinsic to lifelong follow-up: surgical techniques, perioperative management, imaging protocols, and even definitions of key outcomes (e.g., “severe” pulmonary regurgitation or “clinically relevant” arrhythmia) have changed over the last decades, making historical labels imperfectly comparable to current standards. Third, sharing pediatric echocardiography and CMR data across centers is particularly difficult because of strict privacy regulations (GDPR/HIPAA), small sample sizes that facilitate re-identification, and the lack of harmonized consent and governance frameworks.

Technological challenges relate to “black-box” opacity (difficulty understanding why a model yields a given result), performance drift, weak integration with Picture Archiving and Communication Systems (PACS) and EHRs, and high life-cycle costs for retraining and cybersecurity.

Most current ToF-related AI applications still behave as “black-boxes”, providing predictions or segmentations without making their internal reasoning transparent to clinicians. Explainable AI (XAI) techniques can help bridge this gap. For imaging-based models, gradient-weighted class activation maps (Grad-CAM) can be used to highlight the regions of the right ventricular outflow tract, pulmonary valve, or branch pulmonary arteries that drive a given classification (e.g., suspected ToF in fetal or postnatal echocardiography, or the detection of residual lesions on CT/CMR). For perioperative and ICU risk models, Shapley Additive Explanations (SHAP) can quantify how each variable (such as preoperative oxygen saturation, cardiopulmonary bypass time, vasoactive-inotropic score, early arrhythmias, or residual gradients) contributes to the predicted probability of prolonged ICU stay or early complications. Similarly, feature-attribution methods can be applied to long-term risk models of right-ventricular dysfunction, QRS prolongation, or major adverse cardiovascular events, clarifying the relative importance of longitudinal CMR metrics, ECG parameters, exercise capacity, and biomarker trends.

Notably, at least three of the studies summarized in this review already incorporate XAI components: Arnaout et al. [6] used saliency maps and Grad-CAM overlays to visualize which regions of fetal cardiac views drove the classification of complex CHD (including ToF); Wang et al. [10] implemented Grad-CAM within their SOSPCNN CT model to highlight the image regions supporting automatic ToF recognition; and Ishikita et al. [13] applied feature-importance analyses in machine-learning models for 5-year MACE prediction in adults with repaired ToF, identifying the most influential clinical and CMR variables. Taken together, these examples show that XAI is technically feasible across the ToF care continuum and, if adopted more widely, could substantially improve clinical trust and model debugging. Performance drift—the gradual change, usually deterioration, in a model’s predictive performance over time in real-world use, even though the model itself has not been modified—typically arises because the underlying data, clinical practice, or patient population has changed.

Approaches such as privacy-preserving federated learning and realistically simulated synthetic data may help, but they require robust technical and ethical safeguards and coordinated multinational efforts in congenital cardiology.

A short-term roadmap for CHD and ToF should prioritize the creation of federated, multicenter datasets with harmonized labels; pilot use cases such as arrhythmic-risk monitoring and PVR-timing decision support; preregistration of analysis plans; and the implementation of prospective silent trials followed by pragmatic impact studies. Establishing machine-learning operations with version control, bias audits, and post-market surveillance, together with structured human-in-the-loop oversight and training for interdisciplinary teams, will be essential to safely and effectively embed AI into congenital cardiology. Future research should focus on multicenter validation, standardization of AI pipelines, and cost-effectiveness analyses. The involvement of clinicians at every stage of algorithm design is vital to ensure explainability, safety, and real-world relevance. When combined with wearable and imaging data, AI holds the promise of transforming ToF management into a continuous, personalized, and predictive care model, reducing morbidity, optimizing resource use, and improving long-term outcomes. Management of ToF generates highly fragmented data across the life course—ranging from fetal imaging and operative reports to ICU monitoring, longitudinal MRI/echocardiography, and genomic information. AI provides a framework to break down these silos, enabling the construction of continuous, dynamic risk trajectories that recalibrate as new data are incorporated rather than relying on single-episode models. When linked with EHRs and registries, predictive tools can also optimize health-system efficiency by informing the timing of reinterventions, allocating ICU resources, and scheduling advanced imaging during periods of heightened risk. From a research perspective, integrated datasets allow for the discovery of novel phenotypes or endotypes of ToF and can improve the design of clinical trials. Importantly, patient-facing applications—such as mobile platforms offering individualized forecasts and alerts—have the potential to enhance adherence and empower families in shared decision-making.

### Limitations of This Narrative Review

This review is narrative in nature and relies mainly on a PubMed/MEDLINE search complemented by a non-systematic Google search; other bibliographic databases (e.g., Embase, Web of Science, Scopus) and the grey literature were not systematically explored, so some relevant studies may have been missed.

## Figures and Tables

**Table 1:** Comparative summary of artificial intelligence (AI) algorithms, datasets, validation cohorts, and performance metrics used in Tetralogy of Fallot (ToF) diagnosis and management.

Author (Year)	Dataset	Outcome	AI Method (Models)	Training (Validation)	Test (Benchmark)	Best Results	Limitations
Arnaout R. et al. (2021) [6]	prenatal 6000 US studies (normal + complex CHD)	Differentiate normal hearth vs. CHD	DL (Ensemble of CNNs)	1326 US (internal 3-fold cross-validation on the training data)	4000 US from 5 independent datasets, benchmarked head-to-head against 7 fetal cardiology experts using the same five standard views	CHD detection: AUC up to 0.99; Sensitivity 95%, Specificity 96%	Retrospective, single-center training, no external validation
Yu X. et al. (2024) [7]	202 US studies (106 ToF, 96 VSD)	Differentiation between ToF and VSD on fetal utrasound	4 DL (CNNs)	77 VSD + 84 ToF images for training (80/20 split)	19 VSD + 21 ToF images used as an independent test set	AUC 0.873, Sensitivity 85%, Specificity 90.48%, Accuracy 87.8%	Single-center; retrospective; only one view. No external validation, no clinical benchmark
Uus A.U. et al. (2022) [8]	Fetal MRI, 85 research + 93 clinical CHD CMR datasets	Automated localisation, pose estimation and 3D DSVR reconstruction of the fetal thorax	DL + classical registration	32 datasets (318 stacks) for global localization plus 65 datasets (DSVR-reconstructed) for thorax/abdomen and heart/liver landmark segmentation Separate datasets for validation	16 datasets (164 stacks) for global localization and 50 independent stacks from 50 datasets for landmark segmentation benchmarked against manual rigid SVR and manual DSVR on 100 clinical cases	The model achieved ~7 mm localisation error, Dice 0.75–0.85, maintained NCC ≈ 0.41 across 0–180° rotations, and produced clinically acceptable reconstructions in ~88% of early-GA and nearly all late-GA cases	Limited generalisability; sensitivity to artefacts and segmentation errors, which can degrade registration; extreme outliers must be discarded; processing is slow (20–60 min)
Barak-Corren Y. et al. (2025) [9]	CTAs from 42 pts screened for self-expanding TPVR; 76% repaired ToF, 24% PS/PA	CT-based 3D anatomical modeling for pre-procedural risk assessment of coronary compression	DL model trained on prior labeled CTAs	ML model trained on 80 CTAs each with 3D RVOT segmentation	Scans for all 42 pts; additional post-TPVR CTA in 8 pts. Distance tool validated in random 10 cases vs manual 2D measurements Benchmark: limited comparison of 3D distances versus standard manual 2D CT measurements	No significant distance changes (*p* > 0.2); significant systolic shift of closest point (*p* < 0.001); no compression events	Small single-center dataset; no compression events; suboptimal coronary imaging; no biomechanical modeling; unclear added value vs experts
Wang et al. (2021) [10]	Cardiovascular CT scans from 20 children (10 ToF, 10 healthy controls	Diagnostic imaging classification—CT-based recognition of ToF vs healthy controls	custom CNN with stochastic pooling + structural optimization	80 preprocessed CT slices (4 per subject; 40 ToF, 40 HC). Intensive multi-way augmentation; 10 × 10-fold cross-validation on the same dataset (no separate hold-out)	No external test set; performance reported from repeated 10-fold CV on the same 80-image dataset. No clinical benchmark	Sensitivity 92.25 Specificity 92.75 Accuracy 92.50 AUC 0.9587	Very small, single-center retrospective; no external validation or prospective/clinical testing
Lo Muzio F. P. et al. (2021) [11]	Videos from 12 repaired ToF patients undergoing PVR plus 2 additional patients for proof-of-concept testing (1 ToF + good outcome; 1 isolated pulmonary regurgitation + fatal outcome)	Videos of beating RV recorded intraoperatively before and after PVR (86 videos total) for intraoperative RV functional assessment and prognosis prediction	Supervised ML classifiers trained on frequency-domain features from epicardial motion	86 videos (43 pre-PVR = “unhealthy”, 43 post-PVR = “healthy”) from 12 ToF patients; 7 frequency-domain features extracted from x/y motion coordinates; models optimised and 10-fold cross-validated in MATLAB	Qualitative “decision surface” application to 2 additional patients (not in training set) to classify pre/post-surgery videos as healthy vs unhealthy and compare with clinical outcome	AUC ≈ 0.97–0.99. Both models correctly classified the good-outcome ToF patient as unhealthy → healthy and the fatal case as persistently unhealthy, matching prognosis	Very small, single-centre pilot; potential overfitting despite cross-validation, and no real-time prospective trial
Xi L. et al. (2023) [12]	281 ToF children undergoing primary complete repair with CPB	Peri-/post-operative risk prediction: forecasting early clinical adverse events after ToF repair	Supervised ML models	Full cohort with 17 candidate predictors (demographics, echo, operative variables). Random split 4:1 into training and test sets, repeated 5 times	Internal 20% test subsets from same centre (5 random repetitions). No external cohort	Risk factors: CPB time > 116.5 min, RVOT DP > 70 mmHg, TP repair ↑ risk; SpO_2_ < 88% ↑ risk. AUC 0.7 Sensitivity 0.732, specificity 0.716, accuracy 0.721	Retrospective, single-centre cohort; no external validation; relatively modest sample size and event numbers; predictors limited to basic clinical/echo indices
Ishikita A. et al. (2023) [13]	804 repaired ToF adults	Long-term risk stratification: 5-year prediction of major adverse cardiovascular events	Random forest ensemble using 57 clinical, CMR, echo, CPET and lab variables; SHAP for feature importance	235 patients	411 patients	AUC 0.82	Single-centre; requires contemporary CMR (patients with ICD/pacemaker or without CMR excluded)
Ishikita A. et al. (2024) [14]	25 adults with repaired ToF (24% 5-year MACE)	Long-term outcome prediction; comparison of ML vs expert clinicians for 5-year MACE risk	Previously validated random-forest model “AiTOR” using 10 clinical/CMR/CPET variables with SHAP explainability	AiTOR trained on 235 rToF patients with supervised random forest and bootstrap 80/20 splits, then validated in an independent 411-patient cohort	In this study, AiTOR was applied without retraining to 25 rToF patients whose data were also reviewed by five ACHD experts. (Benchmark: direct comparison with individual clinicians and aggregate expert panel; also combined clinician + ML “augmented” scores)	AUC 0.86 vs aggregate experts (AUC 0.92); sensitivity/specificity 83%/79% (AiTOR) vs. 100%/68% (experts). ML support improved AUC significantly on less experienced clinicians (AUC 0.68→0.82, *p* = 0.002)	Very small single-centre sample (25 pts, 5 clinicians), retrospective design, restricted to variables without much missingness, no multi-centre or prospective validation and no incorporation of physical exam/interview data
Samad M.D. et al. (2018) [15]	153 rToF patients with two CMR scans ≥ 6 months apart (median follow-up 2.7 years)	Ventricular deterioration class (none/minor/major) based on change in RVEDVi, LVEF and RVEF between baseline and follow-up CMR	Linear support vector machine classifier with exhaustive feature-subset search and Platt scaling	5-fold cross-validation on the full cohort using 24 baseline clinical/ECG/CMR variables; internal validation via CV only	No separate external test set; performance reported from cross-validated folds within the same 153-patient dataset. (no formal clinical risk-score or human-expert benchmark)	Mean AUC 0.87 (major vs none), 0.82 (any vs none), 0.77 (major vs others), 0.70 (three-class)	Single-centre, relatively small dataset, only internal validation, surrogate CMR endpoints (not hard events)
Mayourian J. et al. (2024) [16]	Repaired ToF	Prediction of 5-year all-cause mortality after an ECG in patients with repaired ToF	12-lead 1D convolutional neural network (AI-ECG) deep-learning model with multi-task output for 1–5-year mortality	Internal test: 1054 pts (13,077 ECGs). Split 95%/5% for training/validation with grid-searched hyperparameters	External validation: 335 TGH pts (5014 ECGs). (Benchmark: Compared against QRS duration and CMR-derived biventricular global function index (BVGFI), plus secondary rToF-specific CNNs)	5-year mortality: AUROC 0.83 (AUPRC 0.18) internal and AUROC 0.81 (AUPRC 0.21) external; AI-ECG AUROC 0.86 vs. QRS 0.69 and BVGFI 0.82 in paired ECG-CMR subset	All-cause (not purely cardiac) mortality, only one external center, thresholds not optimized, no benchmarking vs QRS fragmentation/LGE/BNP, and need for broader multicenter prospective validation
Tilborghs S. et al. (2024) [17]	100 non-ToF ACDC CMR cases + 150 CORRELATE rToF CMRs → 96 ToF for training/validation, 36 ToF internal test, 8 ToF external test (different center/scanner)	Fully automatic LV/RV cavity and myocardium segmentation with ED/ES frame detection and biventricular volume, EF and mass quantification in repaired ToF	3D U-Net–style CNN with marginal Dice loss, multi-class LV/RV cavity + myocardium segmentation on full short-axis stacks	5-fold cross-validation on three configs (non-ToF only, ToF only, mixed); best “Mixed” model (non-ToF + ToF) selected based on TOF validation performance	Ensemble of 5 mixed-training models tested on independent 36-patient ToF test set and 8-patient external ToF set. (Benchmark: compared against commercial CMR package suiteHEART^®^)	On ToF test set: median Dice LV cavity 93.8%/89.8% (ED/ES), RV cavity 92.9%/90.9%, LV/RV myocardium 80.9%/61.8% (ED), RV EDV MAE 12 mL vs. 36 mL with NeoSoft, frame error ≈ 0.6–1 frame	Only ED/ES frames labeled (no quantitative check on intermediate frames), small external cohort, SA-only input (no long-axis), partially labeled data and ToF-only focus so generalisability to other CHD/pathologies and further optimisation still needed
Govil S. et al. (2023) [18]	123 rToF CMRs from San Diego/Auckland for training–validation and 30 rToF CMRs (12 internal, 18 external; multi-vendor 1.5 T) for testing	Fully automated generation of 3D biventricular cardiac shape models (ED/ES) suitable for atlas-based analysis in rToF	End-to-end deep-learning pipeline: ResNet50 (view & slice), CNN-LSTM (phase), U-Net (landmarks) and nnU-Net (myocardial segmentation) feeding a mesh-fitting algorithm	123 Cardiac Atlas Project rToF cases randomly split 111/12 (≈90/10%) per network; internal CV on the 12-case validation set for hyperparameter tuning	Independent 30-patient rToF test set used to compare automated vs manual models. (Benchmarked compared against expert manual contours/landmarks and manually generated shape models including time to create models)	Global MAE between automatic and manual models 1.9 ± 0.5 mm (ED) and 2.1 ± 0.7 mm (ES), SAx cavity Dice ≈ 0.94 and myocardium ≈ 0.78–0.90, Z-score difference ~0.5 SD for first 20 atlas modes	Trained only at ED/ES, modest rToF-only sample, highest errors around valves, some manual overrides still needed, and no evaluation of full-cycle dynamics or clinical endpoints
Van der Ven J.P.G. et al. (2023) [19]	80 pediatric CMR studies (20 healthy, 20 LV-CHD, 20 ToF, 20 univentricular CHD) from three centers, bSSFP short-axis stacks at 1.5 T	Accuracy, reproducibility and time-efficiency of fully automated vs manually adjusted LV/RV segmentation for volumes, mass and stroke volume in children (including ToF)	Two vendor-provided fully automated cardiac segmentation suites (Medis Suite 3.2 and SuiteHeart 5.0) evaluated as black-box algorithms	No training done in this study; algorithms pre-trained by vendors on large adult, structurally normal cohorts with no pediatric/CHD-specific retraining	All 80 pediatric scans used as retrospective test set, comparing fully automated and automated + manual contours against manual reference contours and great-vessel flow SV. Manual expert segmentation (including intra/inter-observer variability) and aortic/pulmonary flow–derived stroke volumes served as benchmarks	Fully automated LV in healthy/LV-CHD showed good agreement (ICC > 0.9) but poorer RV/ToF/univentricular ICCs (down to ~0.6), while automated+manual adjustment yielded ICCs 0.71–1.00 and reduced postprocessing time from ~19–24 to ~5–7 min	Small sample per group, differing age distributions, no absolute gold standard (only manual segmentation and flow), algorithms not trained on pediatric/CHD anatomy, limited ability to analyse subgroups and no Dice/Hausdorff metrics reported
Ložek M. et al. (2024) [20]	Two adolescents with repaired ToF, RBBB, severe PR and RV dysfunction, each with echo, CMR/CT and invasive haemodynamics	To quantify RV electromechanical dyssynchrony and predict effects of pulmonary re-valvulation and RV cardiac resynchronization therapy (RV-CRT)	Patient-specific biophysical “Digital Twin” hearts built with the CircAdapt electromechanical circulation model	Model parameters tuned per patient to match baseline volumes, pressures and RV septal-to-lateral delay; no separate training/validation sets	Virtual experiments simulating PVR, RV-CRT and their combination, compared qualitatively with observed post-intervention changes in the same 2 patients. No formal clinical or computational benchmark; comparison is against each patient’s own measured haemodynamics and strain before and after therapy	Descriptive improvements in modelled RV systolic stretch fraction, wasted work ratio, myocardial work/pump-work ratio and predicted exercise capacity with RV-CRT ± PVR	Only two cases, digital model only approximates patient mechanics (e.g., assumes zero residual delay with CRT), cannot capture long-term remodelling, and provides trends rather than exact quantitative predictions
Marelli A. J. et al. (2024) [21]	19,187 patients with ≥1 CHD, 3784 true CHD by clinician algorithm + manual audit, plus external 68,192-patient cohort (to 2010)	Binary classification of true CHD vs non-CHD in large administrative claims databases	Gradient Boosting Decision Tree (primary), versus SVM, decision tree and regularized logistic regression	80/20 split: 15,400-patient training set with 5-fold CV and grid search to tune GBDT/SVM hyperparameters	Internal 3787-patient test set (725 true CHD) plus independent external 68,192-patient cohort from later years. No human-clinician benchmark	Best GBDT: test AUPRC ~0.994 (train 0.999); external validation accuracy 0.993, F1 0.990, sensitivity 0.978, specificity 0.998	Single-province claims data, reliance on coding quality, possible unrecognized predictors, aggregate (not longitudinal) features and need for replication on other databases
Alkan M et al. (2025) [22]	4153 12-lead ECGs and 595 CPET reports plus clinical letters from 436 adult CHD patients (≈40% ToF) at a single UK ACHD centre	Regression and classification of CPET variables (VE/VCO_2_, VO_2_%pred, VO_2_peak) as surrogate markers of mortality risk in CHD	Covariance-based SVM (and baseline logistic regression) on ECG covariance matrices in Riemannian tangent space, with fusion of bag-of-words clinical-letter features and specialised covariance augmentation	100 repetitions of stratified patient-leave-out splits with SVMs trained on ECG ± clinical-letter features, using internal validation only and ablation of augmentation/fusion strategies	In each repetition, unseen patients formed an internal test set; there was no separate external or prospective test cohort. Benchmark: Compared against models using only vendor/calculated ECG parameters, ECG-only tangent-space features, and versions without Riemannian covariance augmentation or text fusion	Best regression reached r ≈ 0.66 and R^2^ ≈ 0.40 for VO_2_peak, while best classification for VO_2_peak groups achieved accuracy ≈ 0.71, AUC ≈ 0.66 and F1_macro ≈ 0.62	Single-centre, small and highly imbalanced CHD cohort, CPET as surrogate rather than hard outcomes, complex preprocessing, and lack of external validation or direct comparison with clinician judgement

Abbreviations: AI, artificial intelligence; ML, machine learning; DL, deep learning; CNN, convolutional neural network; CMR, cardiovascular magnetic resonance; CT, computed tomography; CTA, computed tomography angiography; US, ultrasound; CHD, congenital heart disease; ToF, Tetralogy of Fallot; rToF, repaired Tetralogy of Fallot; VSD, ventricular septal defect; RV, right ventricle; LV, left ventricle; RVOT, right ventricular outflow tract; TPVR, transcatheter pulmonary valve replacement; PVR, pulmonary valve replacement; CPB, cardiopulmonary bypass; PR, pulmonary regurgitation; PS, pulmonary stenosis; PA, pulmonary atresia; RVEDVi, right ventricular end-diastolic volume indexed; LVEF, left ventricular ejection fraction; RVEF, right ventricular ejection fraction; EDV, end-diastolic volume; ED, end-diastolic; ES, end-systolic; EF, ejection fraction; CPET, cardiopulmonary exercise testing; ECG, electrocardiogram; AI-ECG, artificial intelligence-enabled electrocardiogram; SpO_2_, peripheral oxygen saturation; MACE, major adverse cardiovascular events; MAE, mean absolute error; ICC, intraclass correlation coefficient; SD, standard deviation; SHAP, Shapley additive explanations; SVM, support vector machine; GBDT, gradient boosting decision tree; AUC, area under the receiver operating characteristic curve; AUROC, area under the receiver operating characteristic curve; AUPRC, area under the precision–recall curve; bSSFP, balanced steady-state free precession.

## Data Availability

No new data were created or analyzed in this study; accordingly, data sharing is not applicable to this article.
